# Osteopontin production by TM4SF4 signaling drives a positive feedback autocrine loop with the STAT3 pathway to maintain cancer stem cell-like properties in lung cancer cells

**DOI:** 10.18632/oncotarget.21021

**Published:** 2017-09-18

**Authors:** Soo Im Choi, Seo Yoen Kim, Jei Ha Lee, Jung Yul Kim, Eun Wie Cho, In-Gyu Kim

**Affiliations:** ^1^ Department of Radiation Biology, Environmental Radiation Research Group, Korea Atomic Energy Research Institute, Yuseong-Gu, Daejeon 34057, Korea; ^2^ Department of Radiation Biotechnology and Applied Radioisotope, Korea University of Science and Technology (UST), Yuseong-Gu, Daejeon 34057, Korea; ^3^ Rare Disease Research Center, Korea Research Institute of Bioscience and Biotechnology, Yuseong-Gu, Daejeon 34141, Korea

**Keywords:** cancer stem cells, epithelial-to-mesenchymal transition, lung cancer, osteopontin, TM4SF4

## Abstract

Transmembrane 4 L6 family proteins have been known to promote cancer. In this study, we demonstrated that transmembrane 4 L6 family member 4 (TM4SF4), which is induced by γ-radiation in non-small cell lung cancer (NSCLC) cells, is involved in epithelial-to-mesenchymal transition (EMT) and cancer stem cell (CSC) properties of NSCLC through the regulation of osteopontin (OPN). Forced *TM4SF4* overexpression in A549 cells increased the secretion of OPN, which activates CD44 or integrin signaling and thus maintains EMT-associated CSC-like properties. OPN, known as a downstream target of β-catenin/T-cell factor 4 (TCF-4), was induced by up-regulated β-catenin via TM4SF4-driven phosphorylation of glycogen synthase kinase 3b (GSK3β). TCF4 complexed to promoter regions of *OPN* in TM4SF4-overexpressing A549 cells was also confirmed by chromatin immunoprecipitation. Knockout of either *β-catenin* or *TCF4*-suppressed *OPN* expression, demonstrating that both factors are essential for OPN expression in NSCLC cells. OPN secreted by TM4SF4/GSK3β/β-catenin signaling activated the JAK2/STAT3 or FAK/STAT3 pathway, which also up-regulates OPN expression in an autocrine manner and consequently maintains the self-renewal and metastatic capacity of cancer cells. Neutralizing antibody to *OPN* blocked the autocrine activation of OPN expression, consequently weakened the metastatic and self-renewal capacity of cancer cells. Collectively, our findings indicate that TM4SF4-triggered OPN expression is involved in the persistent reinforcement of EMT or cancer stemness by creating a positive feedback autocrine loop with JAK2/STAT3 or FAK/STAT3 pathways.

## INTRODUCTION

Epithelial-to-mesenchymal transition (EMT) is a cellular process by which epithelial cells lose cell polarity and cell-cell adhesion properties by cytoskeleton reorganization, thus trans-differentiating into mesenchymal cells. Mesenchymal cells decrease adherent junction proteins such as *E*-cadherin and increase *N*-cadherin and vimentin, thus causing morphological changes into fibroblast-like cells [1]. Over the years, EMT has received increased attention because it is associated with pathological states such as cancer progression as well as biological processes including embryonic development [2]. EMT process not only increases the invasiveness and migratory capacity of cancer cells but also provides cancer cells with the ability to evade apoptotic cell death, senescence, and anoikis [3, 4]. EMT is also involved in the acquisition of cancer stem cell (CSC) properties, which lead to chemo- and radio-therapeutic resistance and self-renewal of cancer cells, thereby facilitating tumor recurrence. Tumor cells take advantage of EMT as an intermediary phenotype to achieve self-renewal capacity of CSC [5]. The Tumor microenvironment is also known as a critical regulator of EMT or CSC-like properties that facilitate metastatic dissemination [6]. Because complex networks initiate tumors and maintain EMT-associated CSC-like properties, multiple cellular factors and their underlying molecular mechanisms governing EMT and CSC-like properties should be investigated for potential therapeutic applications. Osteopontin (OPN), one of the multifunctional cytokines, is a highly acidic and glycosylated matrix phosphoprotein secreted in a variety of tissues, including normal bone, dental, renal, and vascular tissues. OPN is involved in a variety of physiological events, including regulation of bone formation, tissue repair, and immune reaction [7–9]. Moreover, it has been recognized as a key component of the EMT-associated pathogenic events in a variety of tumor types. Critical studies showed that OPN binding to CD44 or integrins triggers various signaling pathways, including PI3K/AKT, Janus kinase 2 (JAK2) and the focal adhesion kinase (FAK) pathway, and that OPN functions as a master regulator of EMT [10–12]. Therefore, elevated levels of OPN in the plasma or tumor tissue have been correlated with a poor prognosis for cancer patients [13]. Recent studies have also pointed out important correlations between OPN and CSC phenotypes. OPN has been reported to reinforce stem cell-like properties and to promote γ-radiation resistance in glioblastoma and hepatocellular carcinoma [14, 15].

Transmembrane 4 L6 family member 4 (TM4SF4) is a multi-pass membrane glycoprotein belonging to the transmembrane 4 superfamily, also known as the tetraspanin family, which play roles in the regulation of cell growth, development, and motility [16]. TM4SF4, which has 50% sequence identity with other L6 proteins, is deficient in the characteristic cysteine residue motifs in the EC2 transmembrane domain of the long extracellular hydrophilic loop [17]. Although its structural features imply different functions compared to other members of transmembrane 4 superfamily, there are few studies on TM4SF4 yet except its expression in the liver or intestine [18]. Overexpression of TM4SF4 is shown in hepatocellular carcinoma and colorectal cancer [19, 20] and targeting TM4SF4 with siRNA attenuates the cell growth of hepatocellular carcinoma [21] which implies TM4SF4 is a potential anti-cancer target. In our previous study, we showed *TM4SF4* expression is elevated in non-small cell lung cancer cells (NSCLC) via loss of promoter methylation and confers γ-radiation resistance through activation of the IGF1Rβ/PI3K/AKT/NFκB pathway [22]. Now we show that TM4SF4 is increased by fractionated radiation and its expression is critical for maintaining CSC properties. OPN, a cytokine promoting metastatic and self-renewal capacity, is also increased in fractionated radiation-exposed cells and is shown to be upregulated via TM4SF4. The elevated OPN in lung cancer cells activates STAT3 pathways which stimulate OPN expression. Collectively, we show that TM4SF4 in lung cancer cells mediates the activation of a positive feedback autocrine loop between OPN and STAT3 pathways, resulting in cancer stemness and radiation resistance, and suggest targeting TM4SF4 or OPN may be useful as a cancer treatment.

## RESULTS

### TM4SF4 is up-regulated in ALDH1^high^ as well as fractionated γ-radiation-exposed A549 cells and involved in EMT-associated CSC-like properties

**Figure 1:**
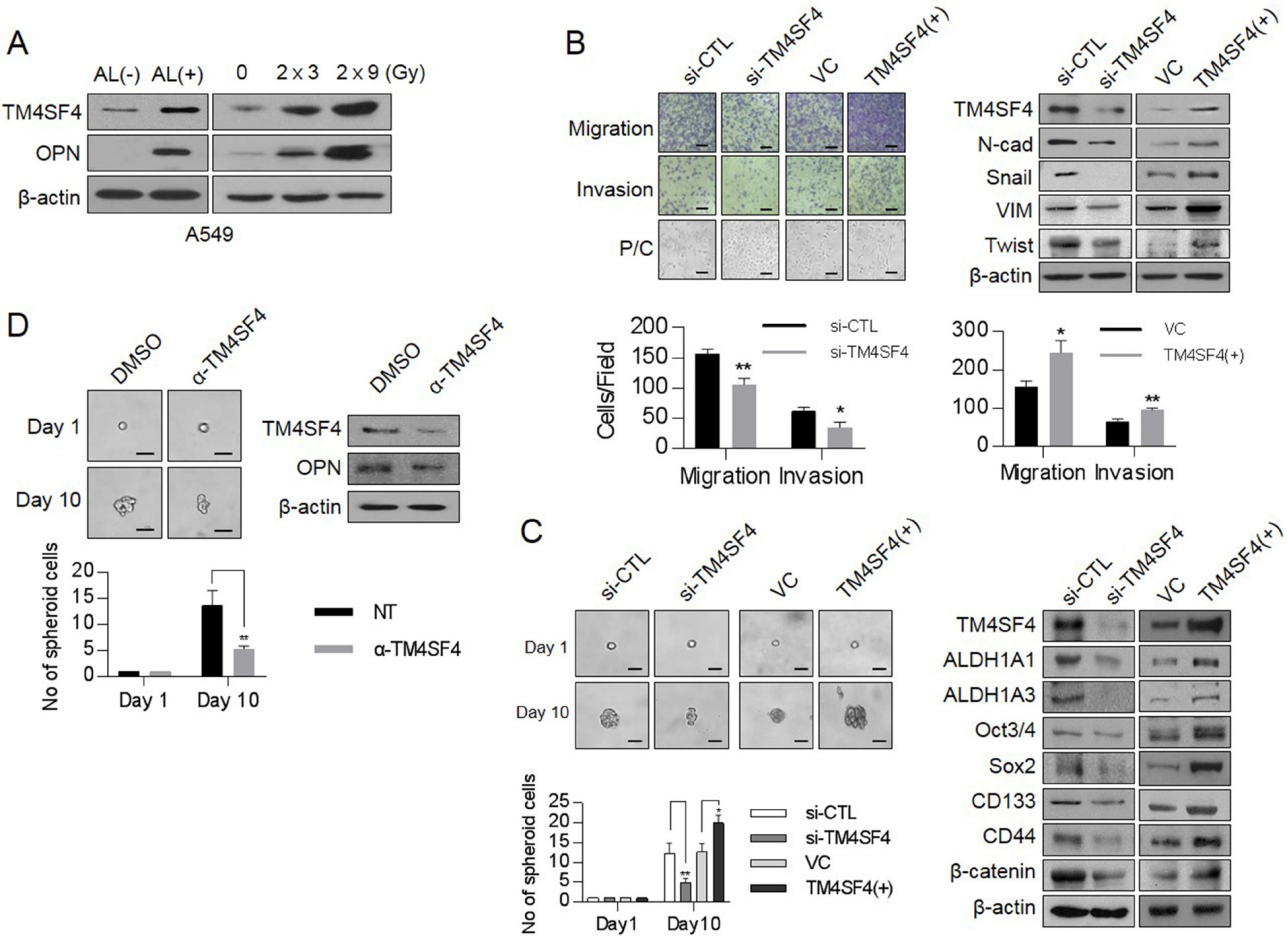
Changes of cellular TM4SF4/osteopontin levels and their related down-stream targets in ALDH1^high^ or fractionated γ-irradiation-exposed cells and control of EMT and CSC properties by TM4SF4 in lung cancer cells (**A**) Western blot analysis of TM4SF4 and osteopontin levels in ALDH1^high^[AL(+)] and ALDH1^low^[AL(–)] cells sorted from A549 cell lines(left panel) and fractionated γ-radiation-exposed cells (2 Gy × 3 times, 2 Gy × 9 times: right panel). (**B**) Changes of migration/ invasion capacity (left panel) and EMT markers including N-cadherin, Vimentin, Snail, and Twist (right panel) in *TM4SF4*-overexpressing or *TM4SF4*-suppressing A549 cells. (**C**) Changes of sphere-forming capacity (left panel) and CSC markers such as Oct3/4, Sox2, CD133, CD44, β-catenin, and ALDH1 (left panel) in *TM4SF4*-overexpressing or *TM4SF4*-suppressing A549 cells. (**D**) Changes of sphere-forming capacity and cellular osteopontin level after treatment with neutralizing antibody against TM4SF4. Data represent the mean of three independent experiments. The quantified results are presented as mean ± s.d. using two-tailed *t*-test. ^*^p < 0.05, ^**^p < 0.01 were considered significant. Scale bar = 20 mm.

Our Previous studies showed that TM4SF4 confers γ-radiation resistance through activation of the IGF1Rβ/PI3K/AKT/NFκB pathway, which is an important signaling pathway in maintaining cancer stemness [22]. We thus questioned whether TM4SF4 is a causative factor that mediates the acquisition of mesenchymal phenotypes and CSC-like properties. Studies on Aldefluor-stained cancer stem cells have demonstrated that ALDH1^high^ cells exhibit increased EMT characteristics with E-cadherin down-regulation and Snail up-regulation [23, 24]. Therefore, cancer cells with high ALDH1 activity are linked to the acquisition of CSC-like properties as well as enhancement of cancer metastasis and resistance to available drug treatments [25, 26]. To study the roles of TM4SF4 in EMT-associated CSC-like cells, A549 NSCLC cells were stained with Aldefluor substrate and sorted to ALDH1^high^ cells and ALDH1^low^ cells (Supplementary Figure 1A). In ALDH1^high^ cells, with the increase of representative stemness marker proteins such as Sox2, Oct4, Notch2, and CD44 (Supplementary Figure 1B), TM4SF4 is also highly up-regulated as compared to counterpart ALDH1^low^ cells (Figure 1A). Simultaneously, OPN, which plays a major role in EMT-associated CSC-like properties of various cancers [13, 27], was up-regulated in ALDH1^high^ cells. Fractionated γ-radiation (2 Gy × 3 times or 2 Gy × 9 times), which enhances EMT and cancer stemness [28], also significantly up-regulated the cellular TM4SF4 and OPN, indicating that these proteins may be involved in the reinforcement of γ-radiation-induced stemness in cancer cells (Figure 1A). To determine whether TM4SF4 is associated with EMT or CSC characteristics, changes in metastatic activity or representative EMT markers were investigated according to *TM4SF4* knockout or overexpression (Figure 1B). *TM4SF4*-suppressing cells with short interfering RNA (siRNA) exhibited down-regulated mesenchymal markers N-cadherin and vimentin. EMT-linked transcription factors such as Twist and Snail are also down-regulated, which is consistent with the effect of TM4SF4 on cellular migration and invasion activity. Moreover, the morphological change from spindle-shaped mesenchymal cells to cobblestone-like cells, a representative biological phenomenon of EMT, was also shown. Forced *TM4SF4* overexpression resulted in opposite effects. Immunocytochemistry staining of TM4SF4 and EMT or stemness markers including vimentin, CD44, and β-catenin confirmed these results again (Supplementary Figure 2). The sphere-forming assay, which evaluates the self-renewal capacity of cancer cells, also showed that TM4SF4 regulates the CSC-like characteristics of A549 adenocarcinoma cells. *TM4SF4* knockdown weakened sphere forming and suppressed the expression of cancer stem cell markers such as ALDH1A1, ALDH1A3, Oct3/4, Sox2. *TM4SF4* overexpression showed exactly the opposite effects (Figure 1C). Moreover, neutralizing antibody treatment to inhibit TM4SF4 action significantly weakened the EMT-associated CSC-like properties of cancer cells with the reduction of the cellular TM4SF4 level (Figure 1D). Neutralizing antibody to TM4SF4 also reduced OPN level, which suggests that TM4SF4 in non-small lung cancer cells may be closely associated with EMT-associated CSC properties via OPN (Figure 1D).

### TM4SF4 promotes EMT-associated CSC-like properties through induction of *OPN* expression

**Figure 2:**
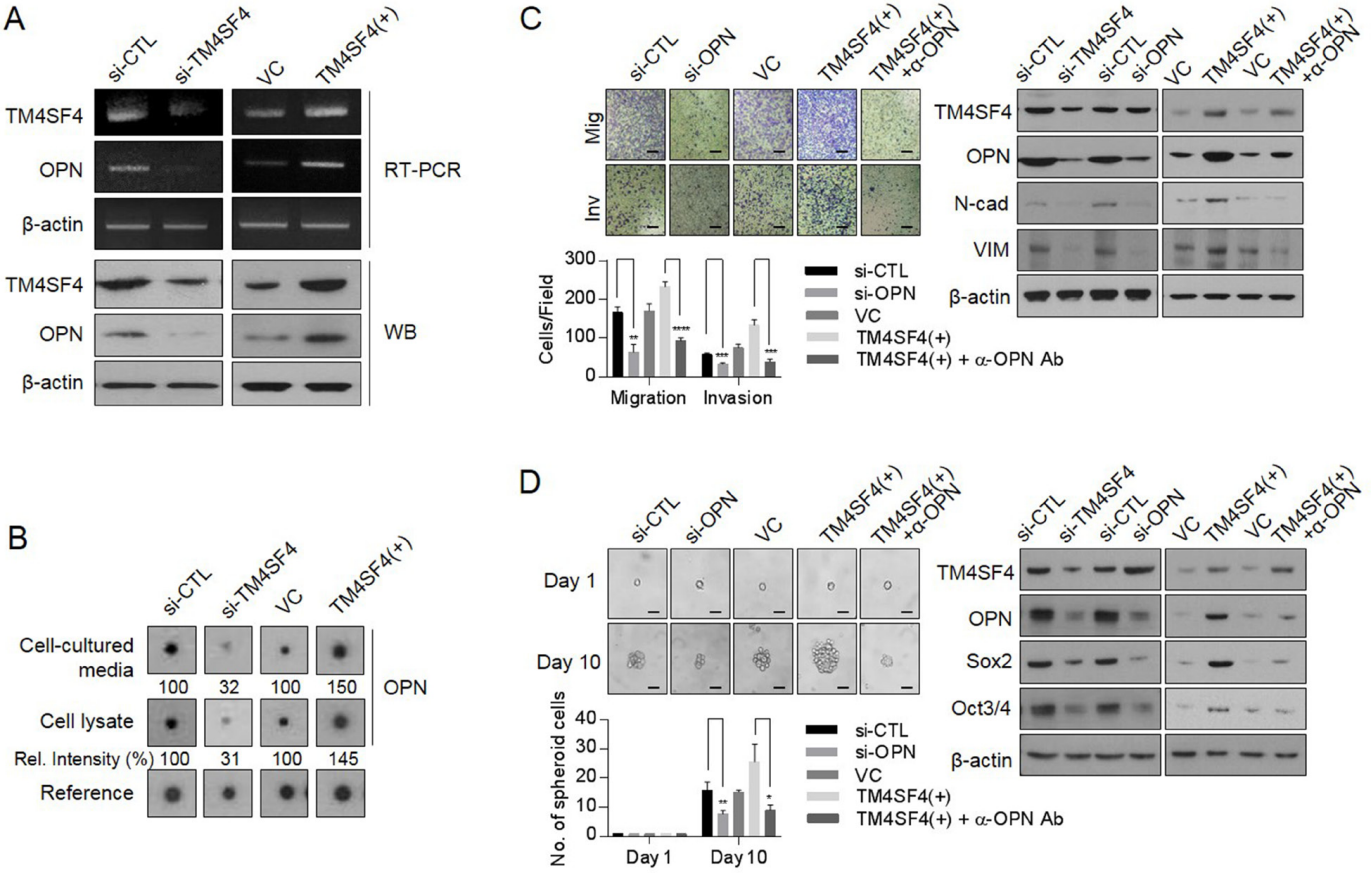
Effect of TM4SF4 on EMT and CSC-like properties through osteopontin secretion in lung cancer cells (**A**) Changes of transcriptional and protein levels of osteopontin in *TM4SF4*-overexpressing or *TM4SF4*-suppressing A549 cells. (**B**) Cytokine array analysis. Changes of osteopontin in cell lysate and cell cultured media of *TM4SF4*-overexpressing or *TM4SF4*-suppressing A549 cells. (**C**) Suppression of migration/invasion capacity (left panel) and EMT markers such as N-cadherin and vimentin (right panel) that were increased in *TM4SF4*-overexpressing A 549 cells. (**D**) Suppression of sphere-forming capacity (left panel) and CSC markers such as Sox2 and Oct3/4 (right panel) that were increased in TM4SF4-overexpressing A549 cells. Data represent the mean of three independent experiments. Scale bar = 20 mm. The quantified results are presented as mean ± s.d. using two-tailed *t*-test. ^*^p < 0.05, ^**^p < 0.01, ^***^p < 0.001, ^****^p < 0.0001 were considered significant.

To investigate whether OPN is involved in promoting EMT-associated CSC-like properties induced by TM4SF4, we examined the cellular levels of OPN according to *TM4SF4* knockout or overexpression. Suppression of TM4SF4 expression with siRNA reduced transcriptional and protein levels of OPN in A549 cells. On the contrary, forced *TM4SF4* overexpression increased OPN (Figure 2A). We also analyzed OPN in cell cultured media that is secreted from A549 NSCLC cells after transfection of siRNA targeting *TM4SF4*. Cytokine arrays showed that secreted OPN was significantly reduced in *TM4SF4*-suppressing A549 cells as compared with control cells. On the contrary, OPN was secreted more in A549 cells with forced *TM4SF4* overexpression, indicating that TM4SF4 is closely involved in *OPN* expression, which can partially reinforce EMT-associated CSC-like properties (Figure 2B). Forced *OPN* knockdown with siRNA significantly weakened the migration and invasion capacity associated with EMT in A549 cells (Figure 2C). Interestingly, treatment with neutralizing antibody against OPN also significantly suppressed the migration and invasion capacity reinforced by *TM4SF4* overexpression in A549 cells. Consistent with these results, neutralizing antibody treatment against OPN decreased cellular levels of mesenchymal marker proteins such as N-cadherin and vimentin, which were up-regulated by forced *TM4SF4* overexpression (Figure 2C). OPN also increased the self-renewal capacity of cancer cells. Down-regulation of *OPN* expression by siRNA significantly inhibited sphere-forming activity (Figure 2D). When cells were treated with neutralizing antibody against OPN, enhanced sphere-forming capacity by forced *TM4SF4* overexpression was also significantly suppressed with the down-regulation of cellular Sox2 and Oct3/4 levels (Figure 2D). These results indicate that OPN is closely involved in the TM4SF4-driven enhancement of EMT-associated CSC-like properties.

### TM4SF4/GSK3β/β-catenin axis is involved in transcriptional regulation of *OPN* expression

**Figure 3:**
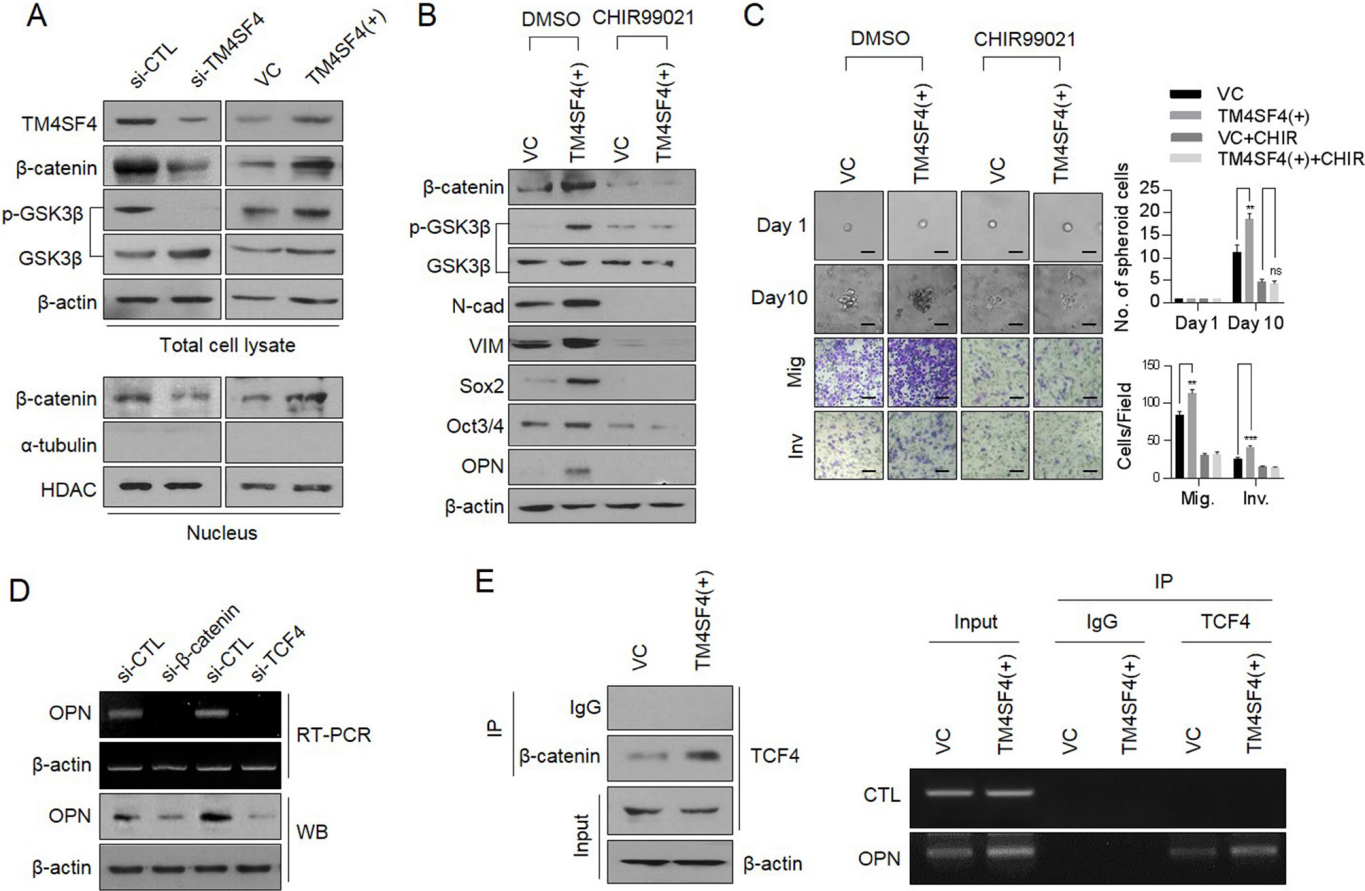
TM4SF4 is involved in transcriptional regulation of osteopontin through the GSK3β/β-catenin signaling pathway (**A**) Western blot analysis of cellular GSK3β (phosphorylation) and β-catenin levels(upper panel) and nucleus fraction β-catenin(lower panel) in *TM4SF4*-overexpressing or *TM4SF4*-suppressing A549 cells. (**B**) Changes of cellular β-catenin, osteopontin, and EMT markers (N-cadherin and vimentin) levels after treatment with CHIR99021 (10μM, 24hr), a GSK3β inhibitor, in *TM4SF4*-overexpressing A549 cells. (**C**) Changes of sphere-forming (upper panel) and migration/invasion capacity (lower panel) in GSK3β inhibitor–treated *TM4SF4*-overexpressing A549 cells. Scale bar = 20 mm. (**D**) RT-PCR and Western blot analysis of transcriptional and protein levels of osteopontin in *β-catenin* and *TCF4-* knockdown A549 cells with siRNA. (**E**) Identification of β-catenin in complexes captured by TCF4 antibody (left panel) and chromatin immunoprecipitation assay between osteopontin promoter region and TCF4 in *TM4SF4*-overexpressing A549 cells (right panel). Data represent the mean of three independent experiments. The quantified results are presented as mean ± s.d. using two-tailed *t*-test. ns = not significant. ^**^p < 0.01, ^***^p < 0.001 were considered significant.

Next, we questioned the signaling network that regulates TM4SF4-stimulated *OPN* expression and secretion in A549 NSCLC cells. β-Catenin, one of the major transcription factors related to EMT in cancer cells [29], was examined. β-Catenin forms a transcriptional complex with T-cell factor 4 (TCF4) as a transcriptional cofactor and induces EMT via Zeb [30]. Accumulation and stabilization of β-catenin are regulated by GSK3β, an important kinase for EMT [31]. GSK3β activation (dephosphorylation) induced destabilization and degradation via phosphorylation of β-catenin. Suppression of *TM4SF4* expression down-regulated the β-catenin levels in the nucleus, as well as overall cellular β-catenin levels and GSK3β, was also activated via dephosphorylation. On the other hand, forced TM4SF4 overexpression resulted in opposite effects (Figure 3A). Treatment of available GSK3β inhibitor, CHIR99021, in *TM4SF4*-overexpressing A549 cells effectively down-regulated β-catenin as well as mesenchymal marker proteins such as N-cadherin and vimentin. CHIR99021 also decreased typical stem cell markers, such as Sox2 and Oct3/4 and reduced OPN (Figure 3B) and clearly suppressed the metastatic and self-renewal capacity of cancer cells (Figure 3C). Suppression of *β-catenin* expression also decreased the cellular level of OPN at the transcriptional and protein levels (Figure 3D). Knockdown of *TCF-4* also suppressed the expression of *OPN* (Figure 3D), which indicates that β-catenin, as well as TCF4, is essential for the *OPN* expression in lung cancer cells. A chromatin immunoprecipitation assay was performed to examine whether the β-catenin/TCF-4 complex directly binds to the promoter of *OPN*. In the immunoprecipitated complexes by neutralizing antibody against TCF-4, the specific promoter region of *OPN* was amplified by RT-PCR and β-catenin complexed with TCF4 was also identified by Western blot (Figure 3E). These results demonstrate that β-catenin participates in the transcriptional regulation of *OPN*expression as a transcriptional cooperation partner of TCF-4 in *TM4SF4*-overexpressing A549 cells.

### TM4SF4 also triggers *OPN* expression through the JAK2/STAT3 or FAK/STAT3 pathway

**Figure 4:**
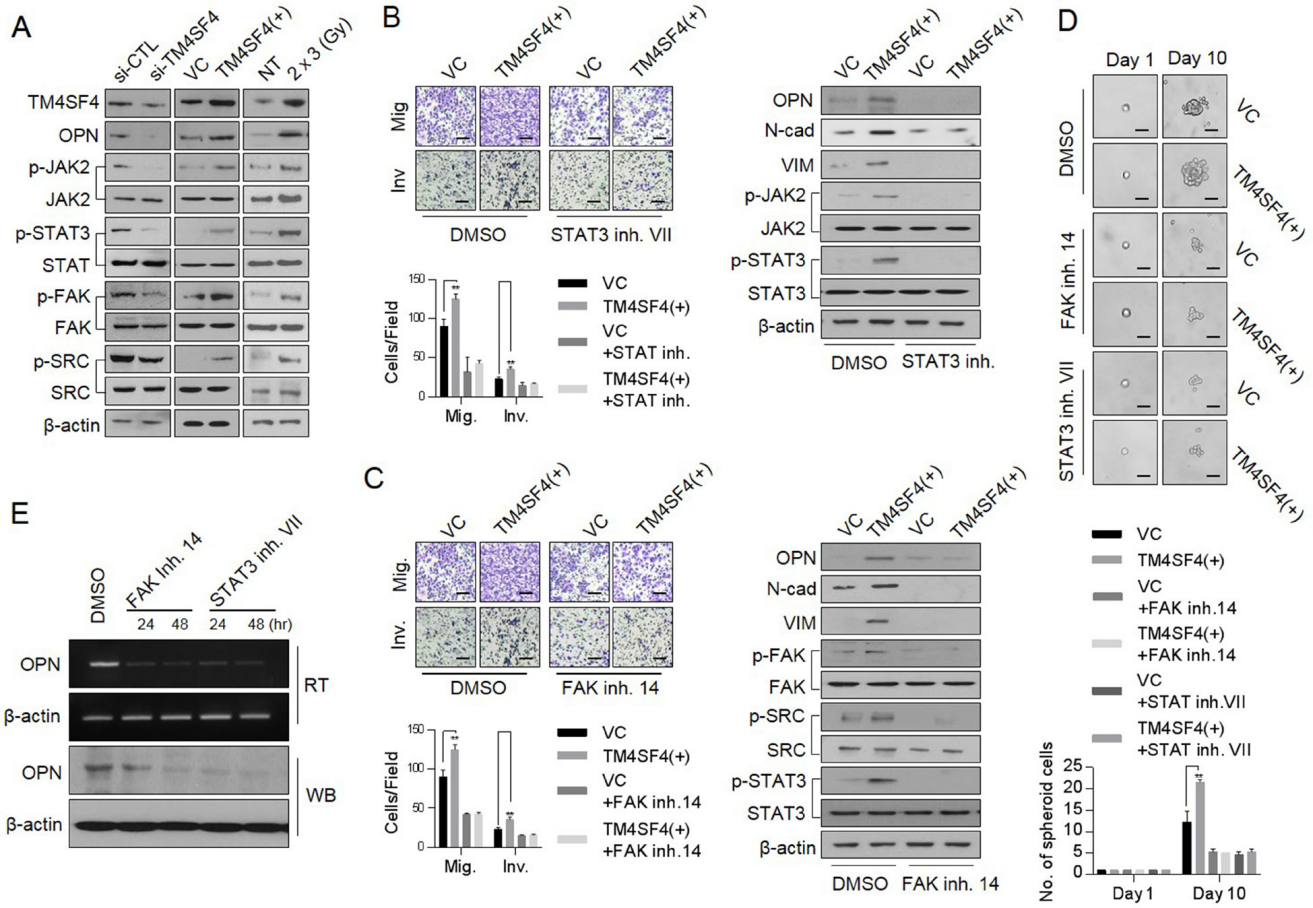
TM4SF4 is involved in the transcriptional regulation of osteopontin via the JAK2/STAT3 or FAK/STAT3 pathway (**A**) Western blot analysis of cellular, p-JAK2, p-FAK, p-SRC and p-STAT3 levels in *TM4SF4*-overexpressing or *TM4SF4*-suppressing A549 cells. (**B**) Changes in metastatic capacity (left panel) and cellular EMT markers (N-cadherin and vimentin) and osteopontin levels (right panel) after treatment of STAT3 inhibitor VII (10 μM, 24 hr), JAK2/STAT3 inhibtior. (**C**) Changes of metastatic capacity (left panel) and cellular EMT markers (N-cadherin and vimentin), p-STAT, p-SRC, and osteopontin levels (right panel) after treatment with FAK inhibitor 14 (10 μM, 24 hr) in *TM4SF4*-overexpressing A549 cells. (**D**) Changes of sphere-forming cellular capacity after treatment of FAK inhibitor 14 and STAT3 inhibitor VII in *TM4SF4*-overexpressing A549 cells. (**E**) Changes of transcriptional and protein levels of osteopontin after treatment with FAK inhibitor 14 and STAT3 inhibitor VII. Scale bar = 20 mm. Data represent the mean of three independent experiments. The quantified results are presented as mean ± s.d. using two-tailed *t*-test. ^**^p < 0.01 were considered significant.

TM4SF4, which is a multi-pass transmembrane protein, apparently mediates signal transduction events that play a role in the regulation of tumorigenesis. Although direct interactors of TM4SF4 have not been identified yet, TM4SF4-mediated activation of cancer stemness or EMT properties is evident as shown above. Therefore, the activation of critical signaling pathways related to tumorigenicity and cancer stemness, including JAK/STAT and SRC/FAK signaling pathway [32–37], were analyzed depending on *TM4SF4* expression. Forced *TM4SF4* knockout with siRNA inhibited the activation of JAK2 and STAT3 (Figure 4A). However, forced overexpression of *TM4SF4* activated JAK2/STAT3 signaling pathway with increments of metastatic capacity (Figure 4A, 4B). TM4SF4 also promoted EMT-associated CSC-like properties through the activation of the SRC/FAK signaling pathway, which sequentially up-regulates activation of STAT3 or AKT. Moreover, fractional radiation on A549 cells, which elevated TM4SF4 as shown above, also activated JAK2/STAT3 and SRC/Fak pathway (Figure 4A). Accordingly, when STAT3 inhibitor VII was treated with forced *TM4SF4*-overexpressing cells, the cellular level of OPN and metastatic activity were also significantly reduced with the down-regulation of mesenchymal marker proteins such as N-cadherin and Vimentin (Figure 4B). Treatment with FAK inhibitor 14 also significantly decreased the cellular OPN, metastatic activity and STAT3 activation as well as down-regulated EMT marker proteins such as N-cadherin and vimentin in A549 cells (Figure 4C). Treatment of either FAK or SATA3 inhibitor decreased not only the metastatic capacity but also the sphere-forming capacity (Figure 4D). Moreover, treatment with both inhibitors (FAK and STAT3 inhibitors) inhibited the transcriptional level as well as the protein level of OPN in spite of TM4SF4-overexprssion, indicating that STAT3 may also function as a potential transcription factor for *OPN* expression (Figure 4E).

### OPN secretion triggered by TM4SF4/GSK3β/β-catenin generates a positive feedback autocrine loop with FAK/STAT3 or JAK2/STAT3 pathway to reinforce OPN production

**Figure 5:**
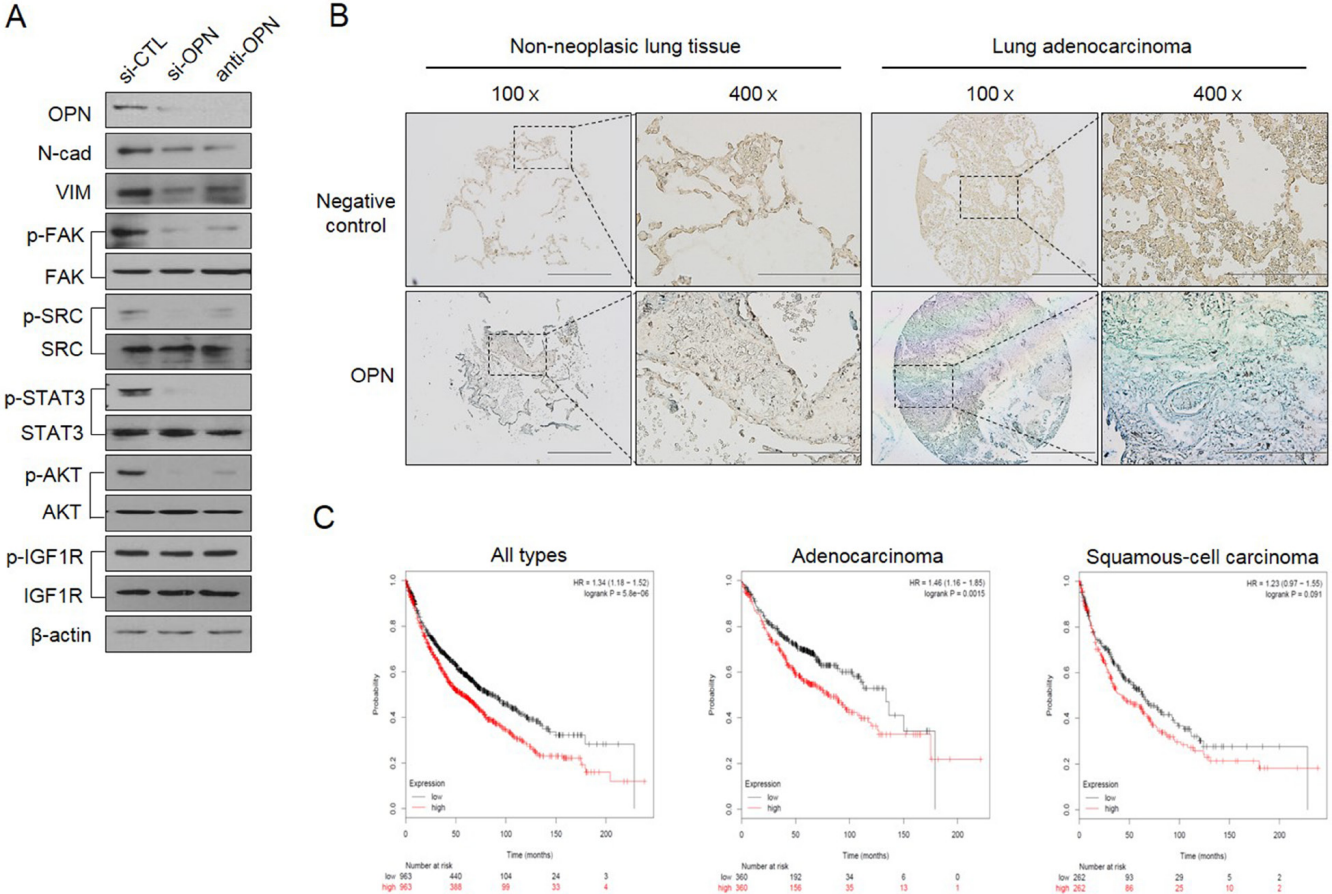
TM4SF4/GSK3β/β-catenin–triggered osteopontin production creates a positive feedback autocrine loop with JAK2 (or FAK)/STAT3 for persistent EMT-associated CSC-like properties (**A**) Changes of cellular EMT marker proteins (N-cadherin and vimentin), p-IGF1Rβ, p-AKT, p-STAT, p-SRC, and p-FAK levels after treatment with siRNA or neutralizing osteopontin antibody. (**B**) Tissue immunostaining and (**C**) Kaplan-Meier survival curves for OPN expression levels in human lung cancer patients. Scale bar = 500 mm (×100), 200 mm (×400).

**Figure 6:**
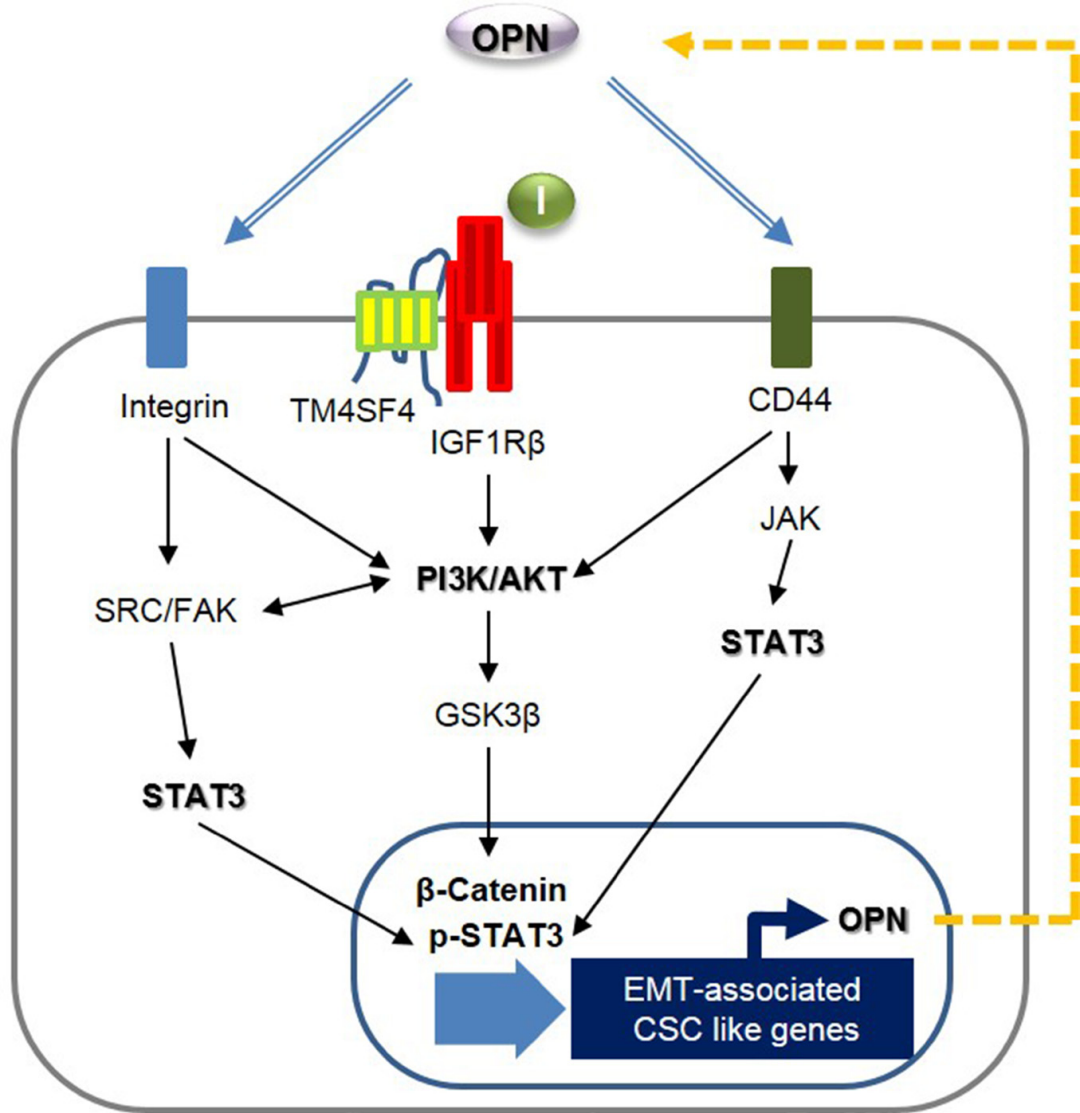
Schematic model illustrating TM4SF4-triggered persistent EMT-associated CSC-like characteristics by a positive feedback autocrine loop between JAK2 (or FAK)/STAT3 and osteopontin

Next, we questioned how TM4SF4 activates the JAK2/STAT3 pathway or SRC/FAK/STAT3 pathway, which also reinforces EMT or CSC-like properties of cancer cells. siRNA-mediated down-regulation of OPN or treatment with neutralizing antibody against OPN blocked OPN binding to its receptors, such as CD44 and integrins, and significantly inhibited the activation of OPN-mediated survival signaling, including FAK, SRC, and STAT3 pathways, consequently weakened EMT-associated CSC-like properties (Figure 5A). Treatment with OPN neutralizing antibody also significantly inhibited PI3K/AKT activation and decreased the cellular β-catenin. However, blocking OPN with neutralizing antibody or siRNA did not inhibit IGF1Rβ activation, suggesting that OPN is involved in multiple PI3K/AKT activation pathways linked to CD44, integrins or FAK, but not to IGF1Rβ, thus promoting the cellular level of β-catenin, although TM4SF4 activates the IGF1R pathway in A549 cells [22]. These results strongly indicate that TM4SF4 significantly drives persistent activation of STAT3 via the positive feedback autocrine loop between the CD44 (or integrins)-mediated JAK2/STAT3 pathway or SRC/FAK/STAT3 pathway and OPN. Finally, Kaplan-Meier (http://kmplot.com/anaysis/index.php?p=service&cancer=lung) and tissue immunostaining analyses showed that with lung cancer, high expression of *OPN* resulted in worse survival rate, indicating that the TM4SF4/OPN axis can be an important potential target for effective cancer therapy (Figure 5B). Based on these results, we suggest that TM4SF4 up-regulates *OPN* expression by triggering the GSK3β/β-catenin signaling pathway and that secreted OPN persistently activates the JAK2/STAT3 pathway or via SRC/FAK/STAT3 pathway autocrine signaling for EMT-associated CSC-like properties in NSCLC cells (Figure 6).

## DISCUSSION

Transmembrane 4 L6 family proteins have been reported to be closely related to cancer malignancy [38]. TM4SF1 is known to promote the self-renewal of esophageal cancer stem-like cells, which is regulated by miR-141; therefore, it was defined as a prognostic marker of squamous carcinomas [39]. TM4SF5 is involved in metastasis via direct activation of FAK, promoting EMT and gefitinib-resistance of cancer cells [40, 41]. In this study, we demonstrated that TM4SF4 is critical for EMT-associated cancer stemness properties in lung cancer cells. TM4SF4-triggered *OPN* expression forms the core for the persistent reinforcement of EMT or cancer stemness by creating a positive feedback autocrine loop with JAK2/STAT3 or FAK/STAT3 pathways. TM4SF4 in NSCLC cells activates also the PI3K/AKT signaling pathway through IGF1Rβ activation and subsequently provided cells with resistance to radiotherapy [22]. The activated PI3K/AKT signals are transferred to GSK3β and β-catenin sequentially. Therefore, treatment of IGF1Rβ inhibitor, AG1024, significantly inhibited phosphorylation of GSK3β, stabilization of β-catenin and consequently *OPN* expression (Supplementary Figure 3), although blocking OPN showed no effects on IGF1Rβ activation (Figure 5A). CD44 or FAK pathways are also associated with AKT activation, thereby contributing to EMT and CSC-like characteristics [35, 42, 43]. These results suggest that TM4SF4-induced GSK3β/β-catenin pathway may be mediated by the activation of the receptor tyrosine kinase (RTK)-linked AKT pathway or non-RTK-linked AKT pathway in lung cancer cells. STAT3 is another critical transcriptional modulator of many solid cancers and is persistently activated in 22–65% of NSCLC cells [44]. Inappropriate activation of STAT3 is closely related to poor prognosis in many cancer patients, including lung cancer patients. JAK is representative up-stream target of STAT3; SRC/FAK is also involved in the activation of STAT3. Collectively, TM4SF4-derived signaling pathways including GSK3β/β-catenin and JAK2(or FAK)/STAT3 appear to be focused on the secretion level of OPN and thus strengthen EMT-associated CSC-like properties in lung cancer cells. We also confirmed that TM4SF4 is associated with *OPN* expression in Calu-3 cells, another NSCLC cells highly expressing *TM4SF4* (Supplementary Figure 4).

Previous reports identified that OPN produced by various cancer cells might promote poor tumor progression in an autocrine manner. OPN is a small integrin-binding ligand protein. At its N-terminal region, OPN has a tripeptide Arg-Gly-Asp (RGD) sequence that interacts with integrins on cell surfaces [45]. Therefore, through interaction with aVb3 integrin, it can trigger metastatic activity or cell growth via activation of the FAK/AKT pathway in lung cancer or activation of the JAK/STAT3 pathway in breast cancer [46, 47]. OPN also functions as a ligand for CD44 [48], hyaluronan receptor, which is overexpressed in cancers with poor prognoses. It interacts with CD44 independent of RGD. OPN binding to CD44 or integrins has been known to up-regulate the expression or activation of various downstream molecules such as MMP-2, MMP-9, cyclooxygenase-2, and vascular endothelial growth factor, which are essential for determining the oncogenic potential of various cancers [49–51]. OPN also increased the tumorigenicity of cancers such as hepatocellular carcinoma in a CD44-dependent manner [52].

Although emerging evidence suggests that STAT3 plays a critical role in EMT and tumor progress, its signaling mechanisms underlying persistent activation of STAT3 associated with EMT-associated CSC-like characteristics remain unclear. Our previous study identified that in breast cancer cell, persistent activation of STAT3 for EMT was partly caused by a positive feedback autocrine loop of PIM-dependent secreted cytokines [53]. Moreover, PKCδ persists with CSC characteristics through an autocrine loop with positive feedback driven by the PKCδ/ STAT3/IL-23/JAK signaling axis [54]. Taken together, we suggest that knockdown of TM4SF4 or OPN significantly may inhibit the self-renewal properties of NSCLC cells and thus TM4SF4 and OPN may be promising targets for CSC therapy.

## MATERIALS AND METHODS

### Cell culture and sphere-forming assay

Human lung cancer cell lines A549 and Calu-3 were obtained from the Korea Cell Line Bank (Seoul, Korea) and were grown in RPMI 1640 or DMEM medium supplemented with 10% (v/v) fetal bovine serum (FBS; Invitrogen, Carlsbad, CA, USA) and 1% penicillin/streptomycin. Cells were incubated at 37°C in a humidified atmosphere of 5% CO_2_. During the sphere-forming assay, cells were suspended in a stem cell–permissive medium including DMEM-F12 (Invitrogen), 20 ng/mL of epidermal growth factor, basic fibroblast growth factor (20 ng/mL) and 2% B27 serum-free supplement (1:50). Then, they were seeded on an ultra-low attachment 96-well plate (Corning Inc., Corning, NY, USA). CHIR99021 (STEMCELL Technologies, Vancouver, BC, Canada), FAK inhibitor 14 (Santa Cruz, CA, USA), and STAT inhibitor VII (Santa Cruz) were used for inactivation of GSK3β, FAK, and STAT3 in the cultured cells respectively.

### Migration assay

The lower culture chamber of a 24-transwell plate (Cell Biolabs, San Diego, CA, USA) was filled with 500 μl migration medium consisting of RPMI 1640 and 10% FBS. Cells were seeded in the upper chamber at a density of 2 × 10^5^ cells in 200 μl serum-free medium/well and incubated for 24 hr at 37°C in a humidified atmosphere of 5% CO_2_. Non-migratory cells in the upper chamber were removed by wiping with a cotton swab. Migratory cells on the bottom of the chambers were stained with crystal violet and cells were counted under a light microscope.

### Invasion assay

Cell invasion was determined using Matrigel-coated invasion chambers (8-μm pores; BD Biosciences, Bedford, MA, USA) according to the manufacturer′s instructions. Cells were resuspended in serum-free RPMI 1640 and placed in the upper invasion chamber at a density of 4 × 10^5^ cells/well. RPMI 1640 containing 10% (v/v) FBS was added to the lower chamber. The plates were incubated at 37°C in a humidified atmosphere of 5% CO_2_ for 24 hr, and noninvasive cells in the upper chamber were removed by wiping with a cotton swab. The invasive cells on the underside of the inserts were fixed with 4% (w/v) formaldehyde in phosphate-buffered saline (PBS) and stained with 2% (w/v) crystal violet in 2% (v/v) ethanol. The stained cells that had penetrated the Matrigel were counted under a light microscope.

### cDNA synthesis and PCR amplification

Total RNA was isolated from cells with TRI Reagent (Molecular Research Center, USA) following the manufacturer's instructions. First-strand cDNA was generated from 1 μg of total RNA using oligo dT primers and a cDNA synthesis kit (Intron Biotechnology, Korea). Resultant cDNA served as templates for PCR amplification with the following primers: TM4SF4 forward, 5′-CCA CGA ATT CAT GTG CAC TGG GGG C-3′ and reverse, 5′-TCC TCG AGT TAA ACG GGT CCA TCT CCC-3′; OPN forward 5′-AGC AGA ATC TCC TAG CCC CA-3′ and reverse 5′-ACG GCT GTC CCA ATC AGA AG-3′; β-actin forward 5′-CAT CCT CAC CCT GAA GTA CCC-3′ and reverse 5′-AGC CTG GAT AGC AAC GTA CAT G-3′ (Table 1). PCR was performed under initial denaturation conditions at 94°C for 5 min, followed by 30 cycles of 94°C for 30 sec, 55°C for 30 sec, and 72°C for 1 min, and then a final extension at 72°C for 5 min. The amplified PCR products were separated on 1% agarose gels (Intron Biotechnology) and stained with ethidium bromide.

**Table 1:** RT-PCR primer sequences

Target	Primer sequence
TM4SF4	F: 5′-CCA CGA ATT CAT GTG CAC TGG GGG C-3′
	R: 5′-TCC TCG AGT TAA ACG GGT CCA TCT CCC-3′
Osteopontin	F: 5′- AGC AGA ATC TCC TAG CCC CA-3′
	R: 5′- ACG GCT GTC CCA ATC AGA AG -3′
β-actin	F: 5′-CAT CCT CAC CCT GAA GTA CCC-3′
	R: 5′-AGC CTG GAT AGC AAC GTA CAT G-3′

F, forward; R, reverse.

### siRNA transfection

A549 cells (1 × 10^5^) were transfected with 50 nM Stealth RNAi^TM^ (Invitrogen) targeting *TM4SF4*, *OPN*(Santa Cruz), *β-catenin* and *TCF4* (Bioneer, Daejeon, Korea) or Stealth RNAi^TM^ Negative Control Medium GC (Invitrogen) using Lipofectamine^®^ RNAiMAX reagent (Invitrogen). Cells were incubated for 48 hr after transfection and then harvested for RT-PCR or western blot analyses. The sequences of Stealth RNAi^TM^ for targeting the genes were as follows: *TM4SF4*; sense, 5´-GCC UCU CAA UGU GGU UCC CUG GAA U-3´ and antisense, 5´-AUU CCA GGG AAC CAC AUU GAG AGG C-3´, *β-catenin*; sense, 5′-CGU UCU CCU CAG AUG GUG U-3′ and antisense, 5′-ACA CCA UCU GAG GAG AAC G-3′, *ΤCF4*; sense, 5′-CAG ACA AAG AAA GUU CGA A-3′ and antisense, 5′-UUC GAA CUU UCU UUG UCU G-3′ , *OPN* (Table 2).

**Table 2:** siRNA sequences

Target	Primer sequence
TM4SF4	S: 5′-GCC UCU CAA UGU GGU UCC CUG GAA U-3′
	A: 5′-AUU CCA GGG AAC CAC AUU GAG AGG C-3′
β-catenin	S: 5′-CGU UCU CCU CAG AUG GUG U-3′
	A: 5′-ACA CCA UCU GAG GAG AAC G-3′
TCF4	S: 5′-CAG ACA AAG AAA GUU CGA A-3′
	A: 5′-UUC GAA CUU UCU UUG UCU G-3′

S, sense; A, antisense.

### Cytokine antibody array and transfection with expression vector

Construction of expression vector and transfections were performed as previously described [18]. Cytokine analysis using cell-cultured media and whole-cell extracts from A549 cells was performed using a human cytokine antibody array membrane kit (Abcam, Cambridge, UK) according to the manufacturer's protocols.

### Western blot analysis

Anti-TM4SF4 antibody (Sigma-Aldrich, St. Louis, MO, USA) was used for Western blot analysis. Antibodies against Sox2, Phospho-FAK(Y397), FAK, Phospho-SRC(Y416), SRC, Phospho-JAK (Y1007), JAK, Phospho-STAT3(Y705), STAT3, TCF4, and CD44 and β-actin antibodies (Cell Signaling Technology, Danvers, MA, USA), Twist, β-catenin (Santa Cruz), E-cadherin, N-cadherin (BD Biosciences), Vimentin (Thermo Fisher Scientific, Fremont, CA, USA), Snail, ALDH1A1, ALDH1A3 (Abcam), Oct4 (Millipore, Billerica, MA, USA), TM4SF4 (Sigma-Aldrich), and Osteopontin (R&D systems, Inc.) were used. The protein concentration was determined with a Lowry kit (Bio-Rad, Hercules, CA, USA). Equal amounts of protein were separated on 8% or 12% sodium dodecyl sulfate–polyacrylamide gel and transferred to a nitrocellulose membrane (Hybond ECL; GE Healthcare Bio- Sciences, Pittsburgh, PA, USA). Blots were blocked for 1 hr at room temperature with blocking buffer (10% nonfat milk in PBS containing 0.1% Tween 20). The membrane was incubated overnight in a cold chamber with specific antibodies. After being washed with Tris-buffered saline, blots were developed with a peroxidase-conjugated secondary antibody and proteins were visualized using enhanced chemiluminescence (ECL) procedures (Amersham ECL reagent; GE Healthcare Bio-Sciences) according to the manufacturer's protocol.

### ALDEFLUOR assay

The ALDEFLUOR assay (STEMCELL technologies, *Vancouver*, BC, Canada) was performed to isolate and characterize CSC populations in A549 cells according to the manufacturer's instructions. Cells (1 × 10^6^) were resuspended in Aldefluor assay buffer containing the ALDH1 substrate. As a negative control, an aliquot of Aldefluor-exposed cells was immediately quenched with diethylaminobenzaldehyde, a specific ALDH1 inhibitor. After 30 min of incubation at 37°C, the cells were washed and sorted as ALDH1^high^ and ALDH1^low^ using a FACSAria flow cytometer (BD Biosciences).

### Immunocytochemistry

Cells (5 × 10^4^ cells/ml) cultured on glass coverslips in six-well plates were fixed with 4% paraformaldehyde and were incubated with the primary antibodies in PBS with 1% bovine serum albumin and 0.1% Triton X-100 at 4°C overnight. CD44 (Cell Signaling Technology, USA), β-catenin (Santa Cruz), Vimentin (Thermo Fisher Scientific), and TM4SF4 (Sigma-Aldrich) antibodies were used. Staining was visualized using an Alexa Fluor 488–conjugated anti-rabbit or Alexa Fluor 555–conjugated anti-mouse antibody (Invitrogen). Nuclei were counterstained using 4, 6-diamidino-2-phenylindole (DAPI; Sigma-Aldrich). Stained cells were visualized with a fluorescence microscope (Olympus BX53F; Olympus, Tokyo, Japan).

### Immunohistochemistry

Lung cancer tissue sections (AccuMax Array; ISU ABXIS Co, Gyeonggi, Korea) were heated at 60°C for 30 min. Sections were deparaffinized and rehydrated by incubation in three changes of xylene (3 min each), three changes of 100% ethanol (2 min each), 95% ethanol for 2 min, 85% ethanol for 2 min, and 75% ethanol for 2 min, and two changes of distilled water. For antigen retrieval, sections were incubated in 0.3% hydrogen peroxide at room temperature for 30 min to deactivate endogenous peroxidase, followed by three washes in PBS. Tissue sections were incubated with anti-OPN polyclonal antibody (1:100; R&D systems) and incubated for 1 hr at room temperature. After three washes with PBS, sections were incubated with secondary reagent (Vectastain ABC Immunohistochemistry Kit; Vector Laboratories, Burlingame, CA, USA).

### Chromatin immunoprecipitation

Chromatin immunoprecipitation (ChIP) assays were performed using the chromatin immuno- precipitation assay kit from Upstate. Cells (1 × 10^7^) were treated with 1% formaldehyde at room temperature for 10 min to form cross-links. Cells were washed twice with PBS and lysed in SDS lysis buffer (1% SDS, 10 mM EDTA, 50 mM Tris, pH 8.1, and protease cocktail inhibitor; Roche) for 10 min on ice. Cells were then sonicated for 5 rounds, each with 10 sec of sonication, with 1 min of rest between rounds to shear chromatin. Samples were divided into three parts: input, TCF4 antibody, and serum(IgG). TCF4 Ab and IgG samples were precleared with salmon sperm DNA/protein agarose-A beads. TCF4 Ab samples were exposed to precipitating antibody (5 μg) overnight. After immunoprecipitation, immune complexes were collected by adding salmon sperm DNA/protein agarose-A beads. Immune complexes were washed and eluted and the cross-links were reversed by heating. Precipitated DNA was recovered using Proteinase K digestion, phenol extraction, and ethanol precipitation. The OPN promoter regions were PCR-amplified from the TCF4 Ab, IgG, and input DNA samples. The OPN promoter regions used the following primers: forward 5′-GCC TAA GGC AAC AGA GC-3′ and reverse 5′-TCC AGC GGG ATA GAA CAC TC-3′, control primers: forward 5′-ATG GTT GCC ACT GGG GAT CT-3′ and reverse 5′-TGC CAA AGC CTA GGG GAA GA-3′. Recovered DNA was amplified using 34 amplification cycles (94°C 1 min, 55°C 1 min, 72°C 1 min). Assays were repeated three times to confirm the reproducibility of PCR results.

### Preparation of nuclear/cytoplasmic fractions

Cells were washed with ice-cold PBS, scraped from the plate, and centrifuged (1000 rpm, 5 min, 4°C). The cell pellet was resuspended in a lysis buffer (10 mM Tris/HCl, pH 7.4, 10 mM NaCl, 3 mM MgCl_2_, 0.5% Nonidet P40, and protease inhibitors) and then applied to 6 ml of sucrose buffer (0.7 M sucrose, 60 mM KCl, 15 mM NaCl, 15 mM Tris/HCl, pH 7.5, 2 mM EDTA, 0.5 mM EGTA, 14 mM 2-mercaptoethanol, and 0.1% Triton X-100). After 10 min of centrifugation (3500 rpm, 4°C), the cytoplasmic fraction was harvested from the top of the sucrose buffer, and the nuclei forming a pellet at the bottom of the tube were lysed in RIPA buffer (50 mM Tris/HCl, pH 7.4, 150 mM NaCl, 0.5 mM EDTA, 1% Triton X-100, 0.5% deoxycholate, 0.1% SDS, 5 μg/ml DNase and protease inhibitors) for 20 min on ice. Then, cell lysates were processed as described with centrifugation in the sucrose buffer. The purity of fractions was tested by Western blotting for α-tubulin as a cytoplasmic marker and HDAC1 as a nuclear marker.

### Statistical analysis

Statistical analysis was performed using PRISM version 5.0 (GraphPad, San Diego, CA, USA). Results are shown as the mean ± s.d. from at least 3 experiments. The two-tailed *t*-test was used for statistical comparisons between groups; *p* < 0.05 was considered statistically significant (^*^p < 0.05, ^**^p < 0.01, ^***^p < 0.001, ^****^p < 0.0001 versus control).

## SUPPLEMENTARY MATERIALS FIGURES AND TABLES


