# Supplementary material for: Osteopontin production by TM4SF4 signaling drives a positive feedback autocrine loop with the STAT3 pathway to maintain cancer stem cell-like properties in lung cancer cells
Source: Oncotarget. 2017 Sep 18;8(60):101284–97. doi: 10.18632/oncotarget.21021 (PMC5731874; doi:10.18632/oncotarget.21021)
Supplement: Supplementary file 1 [file oncotarget-08-101284-s001.pdf]

Osteopontin production by TM4SF4 signaling drives a positive feedback autocrine loop with the STAT3 pathway to maintain cancer stem cell-like properties in lung cancer cells

SUPPLEMENTARY MATERIALS

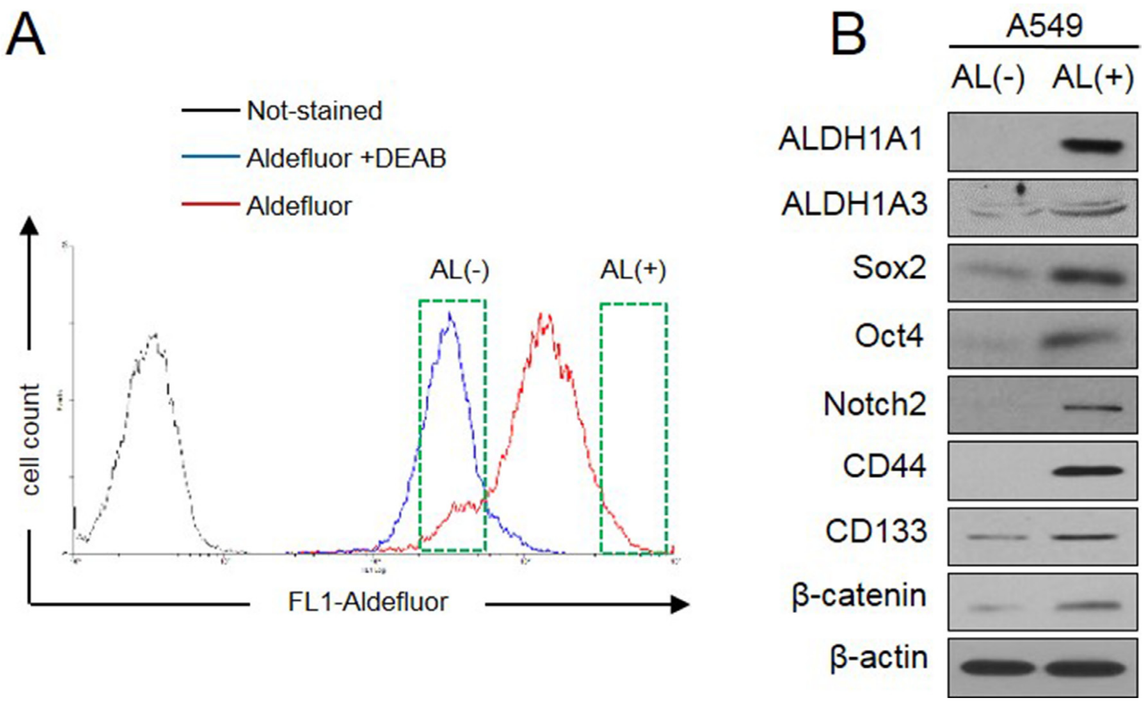

Supplementary Figure 1: ALDH1^{high} and ALDH1^{low} cells were sorted from A549 cells. As indicated in the histogram, ALDH1^{high} cells[AL(+)] and ALDH1^{low} cells[AL(–)] were gated and sorted using an ALDOFLUOR™ reagent and FACS Aria (BD Biosciences) (**A**) and stemness markers such as Sox2 and Oct4 was analyzed by Western blot (**B**).

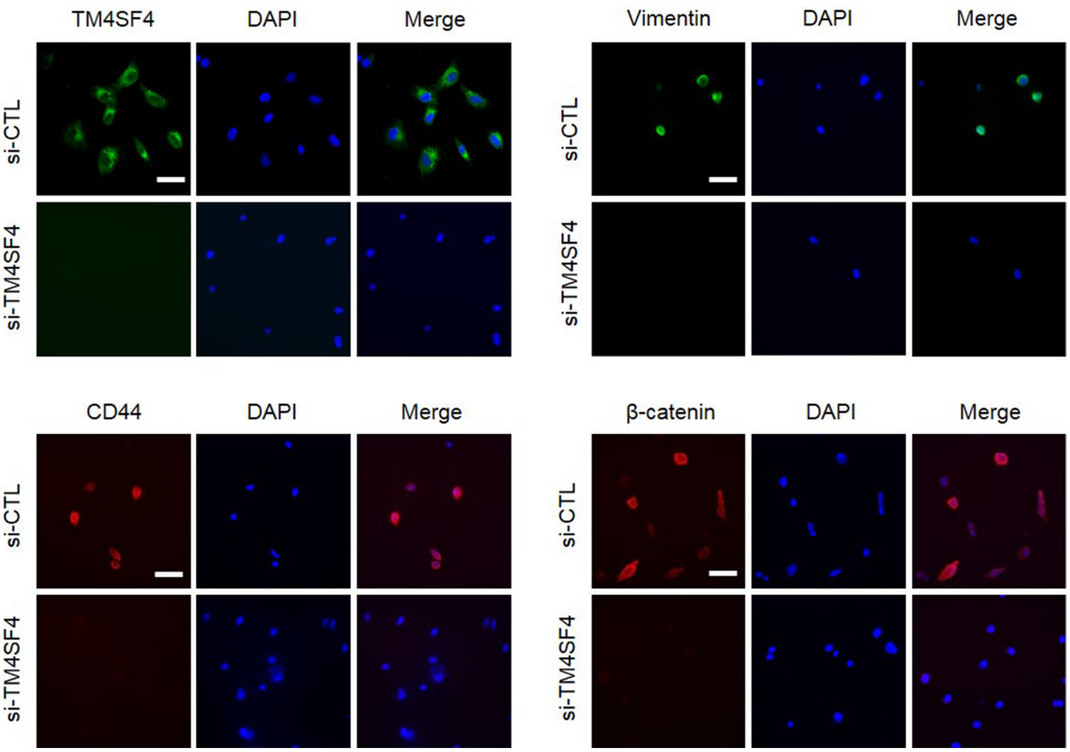

Supplementary Figure 2: Immunostaining of TM4SF4, vimentin, CD44 and β-catenin in *TM4SF4*-suppressing A549 cells.

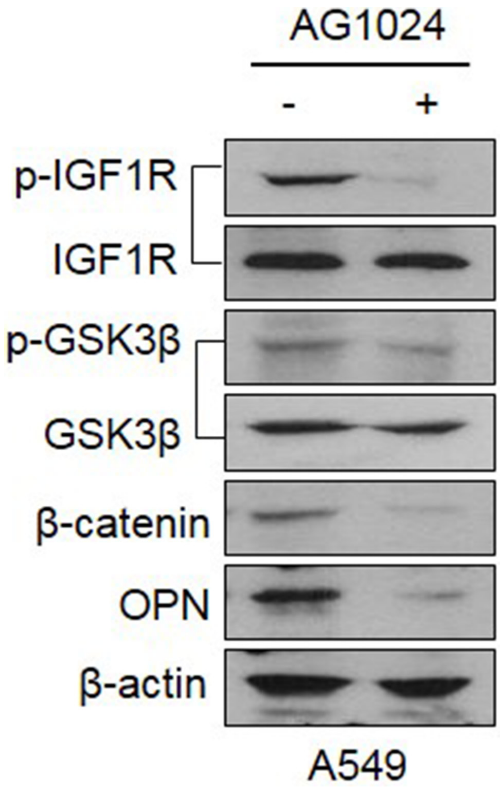

Supplementary Figure 3: Changes in cellular levels of GSK3β, β-catenin, and osteopontin after treatment with IGF1Rβ inhibitor, AG1024 (10 µM, 24 hr).

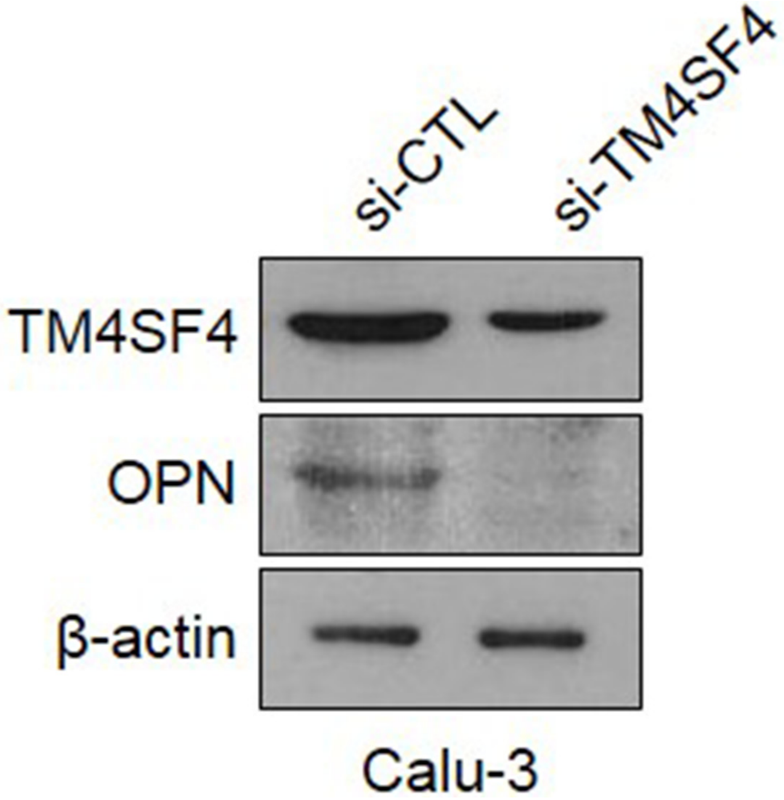

Supplementary Figure 4: Changes in cellular osteopontin level in *TM4SF4*-suppressing Calu-3 NSCLC adenocarcinoma cells.
